# Evaluation of the Effect of Light Color on Albumins and Globulins Content During Bean Germination

**DOI:** 10.3390/foods14101750

**Published:** 2025-05-14

**Authors:** Victor Manuel Rivera Aguilar, José Pedraza-Chaverri, David Julian Arias-Chávez, Ruth Jaimez, Edgar Flores-Soto, Isaías E. Garduño, Fernando S. Chiwo, Celia Sánchez Pérez, Ana del Carmen Susunaga Notario

**Affiliations:** 1 Facultad de Química, Universidad Nacional Autónoma de México, Circuito Exterior S/N, Ciudad Universitaria, Coyoacán, Mexico City 04510, Mexico; 316325628@quimica.unam.mx; 2 Departamento de Biología, Facultad de Química, Universidad Nacional Autónoma de México, Circuito Exterior S/N, Ciudad Universitaria, Coyoacán, Mexico City 04510, Mexico; pedraza@unam.mx; 3 Farmacología y Propiedades Terapéuticas de los Alimentos, Escuela de Dietética y Nutrición del ISSSTE, Tlalpan, Mexico City 14070, Mexico; david.arias@ednissste.com.mx; 4 Departamento de Farmacología, Facultad de Medicina, Universidad Nacional Autónoma de México, Circuito Exterior S/N, Ciudad Universitaria, Coyoacán, Mexico City 04510, Mexico; ruth.jaimez@facmed.unam.mx (R.J.); edgarfloressoto@yahoo.com.mx (E.F.-S.); 5 Secihti—CIATEQ A.C., Eje 126 No. 225, San Luis Potosí 78395, Mexico; isaias.garduno@ciateq.mx; 6 Escuela de Creatividad, Universidad Marista de San Luis Potosí, Av. Beato Marcelino Champagnat 305, San Luis Potosí 78183, Mexico; 1958@umaslp.maristas.edu.mx; 7 ICAT Instituto de Ciencias Aplicadas y Tecnología, Universidad Nacional Autónoma de México, Circuito Exterior S/N, Ciudad Universitaria, Coyoacán, Mexico City 04510, Mexico; celia.sanchez@icat.unam.mx; 8 Secihti—ICAT Instituto de Ciencias Aplicadas y Tecnología, Universidad Nacional Autónoma de México, Circuito Exterior S/N, Ciudad Universitaria, Coyoacán, Mexico City 04510, Mexico

**Keywords:** germination, bean black, light, color, albumins and globulins

## Abstract

The effect of different light colors on the concentration of albumins and globulins during black bean (*Phaseolus vulgaris* L.) germination was evaluated with an RGB LED lighting system. This study aimed to determine how light of different spectral composition influences protein content across different germination stages. Black bean seeds were germinated under six different LED light sources (red, green, blue, white, violet, and cyan), and protein fractions were quantified by Bradford’s method. The results showed that blue and cyan light increased the concentration of albumins. Blue, white, and cyan light for globulins increased the concentration compared to germination under solar conditions for both fractions. The positive correlation between these protein fractions under specific wavelengths suggests a metabolic adaptation to light exposure. These findings highlight the potential of controlled lighting conditions to enhance the nutritional quality of germinated beans, supporting their application as functional food ingredients. Additionally, this study underscores the importance of photobiological modulation in seed germination, opening new possibilities for optimizing plant-based protein sources. Future research could explore the mechanisms behind these protein variations and their implications for food production and nutrition.

## 1. Introduction

Beans are one of the most important staple foods in Mexican cuisine. As a crop native to Mexico, beans hold significant historical and cultural value. They have been integral to the country’s gastronomy since pre-Hispanic times. Their cultivation has a considerable economic impact due to their production, commercialization, and employment generation [1,2].

Among the different varieties, black beans (*Phaseolus vulgaris* L.), belonging to the Fabaceae family, are herbaceous plants native to Central and South America. They exhibit annual flowering, a climbing growth habit, and stems that may be either pubescent or glabrescent at maturity [3]. Black beans are characterized by their pod-shaped fruit, which contains seeds classified as legumes. This crop is highly adaptable to diverse agroclimatic conditions, thriving mainly in temperate to warm climates with well-drained soils and moderate moisture levels [4].

Black beans are known for their high nutritional value. They contain important molecules with high activity in human metabolism, such as simple and complex carbohydrates, dietary fiber, protein, polyphenols, and minerals. It has been shown that the consumption of this legume is associated with health benefits, for example, in the reduction in glycemic index and low-density lipoproteins (LDL) [5,6,7,8,9]. Their high protein content represents approximately 70% of the protein quality compared to a reference animal protein, which is considered 100% quality, primarily taking into consideration the essential amino acids [10]. This gives them a high nutritional value. Their consumption and combination with cereals have been considered optimal to cover the recommended daily intake (RDI) [11]. In addition, peptides derived from bean proteins have antioxidant activity, which can contribute to the prophylaxis and treatment of various diseases [12].

The protein contribution of black beans is determined by the sum of all their protein fractions. According to Osborne’s classification (1924), the most abundant proteins present in black beans are albumins and globulins [13,14].

Bean albumin and globulin fractions possess techno-functional properties that make them highly applicable in the food sector. These include water- and oil-holding capacity, stable foam and emulsion formation, and high solubility over a wide pH range [9,15].

Germination is the process by which a seed gives rise to a new plant. It begins with water absorption by the dry, dormant seed, followed by the elongation of the embryonic axis, culminating in the seed coat rupture and radicle emergence [16,17,18]. Mechanically, germination results from the interaction between two opposing forces: the embryo’s growth potential and the resistance imposed by the seed coat [18]. This process involves transforming cells from a dehydrated state with low metabolic activity to a hydrated and metabolically active condition. During germination, cells transition from a dehydrated, metabolically inactive state to a hydrated and metabolically active condition. The study of these mechanical and flow properties, particularly during water uptake and cell expansion, falls under the domain of rheology. Rheological changes during germination are critical, as they influence the seed’s ability to absorb water, mobilize nutrients, and facilitate cell wall loosening, all essential for successful germination [19].

Water uptake occurs in three different and consecutive phases: (1) imbibition, characterized by water absorption without apparent morphological changes; (2) germination sensu stricto, during which reserve substances such as proteins, carbohydrates, and polyphenols stored in the cotyledons are hydrolyzed by enzymes such as amylases, proteases, and lipases, providing metabolic energy to the seedling until it becomes capable of photosynthesis; and (3) embryo growth or radicle emergence, marked by a shift in the physiological state of the embryo. The regulation of this process is primarily mediated by hormones and gene expression, with abscisic acid (ABA), gibberellins (GA), and the DELAY OF GERMINATION 1 (DOG1) gene playing key roles [20,21,22,23].

Seed germination potential or vigor is a complex physiological trait that ensures the rapid and uniform radicle emergence and seedling establishment. For effective germination, optimal environmental conditions must be met, including appropriate moisture, temperature, pH, air composition, and light availability [24]. Light is fundamental in regulating germination, particularly through the activation of photoreceptors that transform light energy into biochemical energy. Notable examples include cryptochromes and phototropins, which respond to blue light, and phytochromes, activated by red light [25]. This activation subsequently regulates the biosynthesis of GA and ABA, either promoting or inhibiting the germination process [23]. Germination responses can be modulated by visible light, depending on its spectral composition within the 400–700 nm range, with specific effects determined by their spectral components. In this context, red, green, and blue (RGB) LED light sources generate three primary optical colors, producing different luminous stimuli: the combination of R+G+B results in white light, R+B produces violet light, and G+B generates cyan light. The effect of each light color on the concentration of protein fractions during black bean germination is unique and is associated with the dynamic protein metabolism of the seed [26].

Over the past decades, research has focused on increasing the concentration of nutrients in various seeds through the germination process [7]. As a result, germinated seeds such as soybeans, wheat, lentils, alfalfa, and barley have been classified as functional foods. These can be consumed as ready-to-eat (RTE) products, used as wheat flour substitutes in baked goods, incorporated into nixtamalized products, or employed as raw materials to produce plant-based products [27,28,29,30,31].

Therefore, the objective of this study is to evaluate the effect of different light colors on the concentration of albumins and globulins during the germination of black beans (*Phaseolus vulgaris* L.). Specifically, we aim to determine whether exposure to distinct light wavelengths during germination can enhance the protein profile of the sprouts, thereby improving their functional properties—such as solubility, water and oil absorption capacity, emulsifying and foaming abilities—and expanding their potential applications in the development of functional foods and plant-based protein sources.

## 2. Materials and Methods

Black bean seeds used for germination were purchased from a supermarket in Mexico City, and the product origin was determined from commercial labeling. A glass tray was used as the germination support, prepared with a cotton bed, 50 g of coconut fiber, and an additional cotton layer on top. The germinated seeds were dried in a food dehydrator (Hamilton Beach 32100^®^), and flours were obtained for each developmental stage using a mortar or grinder. The lighting system used was an RGB LED strip from STEREN^®^.

Sigma Aldrich (St. Louis, MO, USA) provided the albumin standard from chicken egg white, Bradford reagent, and sodium chloride (≥98% purity). Spectrophotometric measurements were performed with an UV-Vis-NIR spectrophotometer.

Spectral composition of RGB LED colors

Emission spectra of six RGB LED light colors: red, green, blue, white, violet and cyan used in the study were registered using a FLAME UV-Vis A UV-Vis-NIR spectrophotometer and the DH 2000 light source Ocean Optics^®^.

Seed conditioning and bean germination

The seeds were cleaned to remove foreign material and washed with running water. Activation: seeds were soaked in purified water for 12 h. Germination sensu stricto: The germination experiment lasted seven days. Thirty equidistant seeds were placed on the hydrated support (pre-treated with 300 mL of purified water). Every 8 h, 50 mL of purified water was automatically supplied to each tray throughout the experiment. The trays were exposed to LED RGB light colors in the following order: primary colors (red, green, and blue) and their combinations (white, violet, and cyan). For all trials, the lights were placed on a board 20 cm away from the seeds so that the LED strip light would allow uniform illumination. The maximum intensity level was set according to the manufacturer’s specifications. The LED light strip was replaced for each color evaluated to rule out variations due to wear and tear.

Classification of developmental stages

The seeds were classified into five stages: (1) imbibition, (2) non-visible radicle, (3) visible radicle, (4) root and (5) seedling.

Flour preparation

Each sample was dried at 40 °C for 48 h in a food dehydrator (Hamilton Beach 32100^®^), and flours were obtained using a mortar or coffee grinder.

Extraction and quantification of albumins and globulins

Extraction was performed separately for each protein fraction. A total of 15 mg of flour was weighed, and 1 mL of extraction solvent was added: distilled water for albumins and 0.5 M NaCl solution for globulins. The samples were stirred for two hours and centrifuged at 2500 rpm for 15 min [13,14]. The supernatant was collected, and aliquots were taken for quantification using the Bradford method, with ovalbumin as the standard and absorbance measured at 595 nm [32].

Percentage increase and decrease in albumins and globulins

The increase in protein concentration (% Increase and decrease) in germinated seeds was calculated relative to black bean flour concentration, assigning a 100% value to the latter as a reference of protein content of ungerminated seeds. The calculation was performed for each stage and light exposure condition using the following Equation (1).

(1) $$\%\;Increase\;and\;decrease=\frac{\left(C_{X}-C_{100,i}\right)}{\left(C_{100,i}\right)}\times 100$$

*C_100,i_* represents the protein concentration for *i* = 0 for ungerminated seeds (US) as negative control, and *i* = 1 for seeds germinated under sunlight as positive control (100% value), while *Cx* is the concentration in seeds germinated under different light colors.

Statistical analysis

Values were expressed as mean ± standard error of the mean (SEM). Significant differences between groups were determined using one-way or two-way analysis of variance (ANOVA). Post hoc comparisons between groups were conducted using Tukey or Dunnet tests with GraphPad Prism 8.0 software (GraphPad, San Diego, CA, USA). Results were considered significant for *p* < 0.05.

## 3. Results and Discussion

### 3.1. Emission Spectra of RGB LED Sources

**Figure 1:**
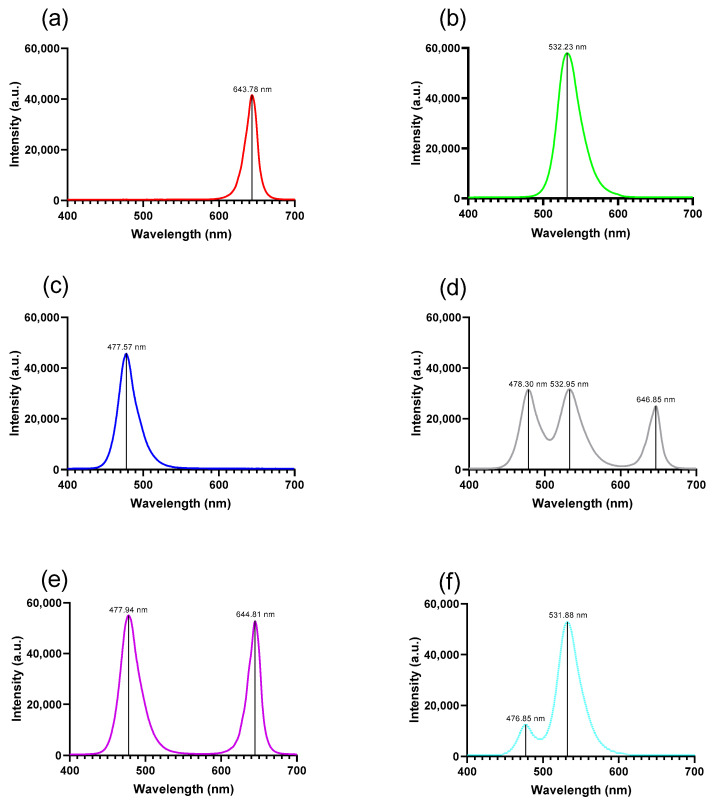
Emission spectra of the RGB LED light colors used during germination tests for: (**a**) red light. (**b**) green light. (**c**) blue light. (**d**) white light. (**e**) violet light and (**f**) cyan light. nm = nanometers; a.u. = arbitrary units.

The effect of spectral illumination on protein concentration during germination was evaluated in different regions of the electromagnetic spectrum. We measured the transmission spectra for six LED sources. Figure 1 presents the emission spectrum with the maximum emission for each light color.

### 3.2. Seed Selection and Germination

**Figure 2:**
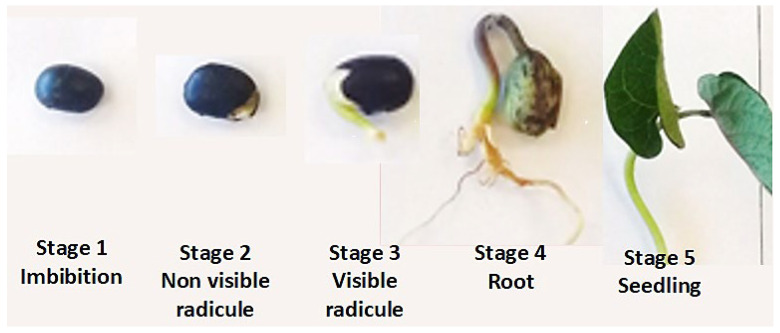
Germination stages of black bean (*Phaseolus vulgaris* L.) seed.

This study proposed five germination stages, covering the sensu stricto germination phase, where the highest metabolic mobilization occurs. Germinated black bean seeds were classified based on their morphology on day 7. In stage 1, seeds maintained their active form (imbibition). Stage 2 included seeds with non-visible but tactilely perceptible radicles. In stage 3, the radicle emerged; in stage 4, root development was observed, and in stage 5, seedlings fully developed (Figure 2).

Seeds were soaked in purified water for 12 h before germination to achieve imbibition (the first phase of germination). This process is intrinsically linked to light perception by photoreceptors such as phytochromes and cryptochromes, as dry seeds are less efficient at detecting light than hydrated ones due to their need for free water for optimal function [33]. These receptors induce internal signaling as a direct effect of light reception and regulate germination through the biosynthesis and catabolism of hormones like ABA, which inhibits germination (dormancy), and GA, which promotes germination [23,34,35].

**Figure 3:**
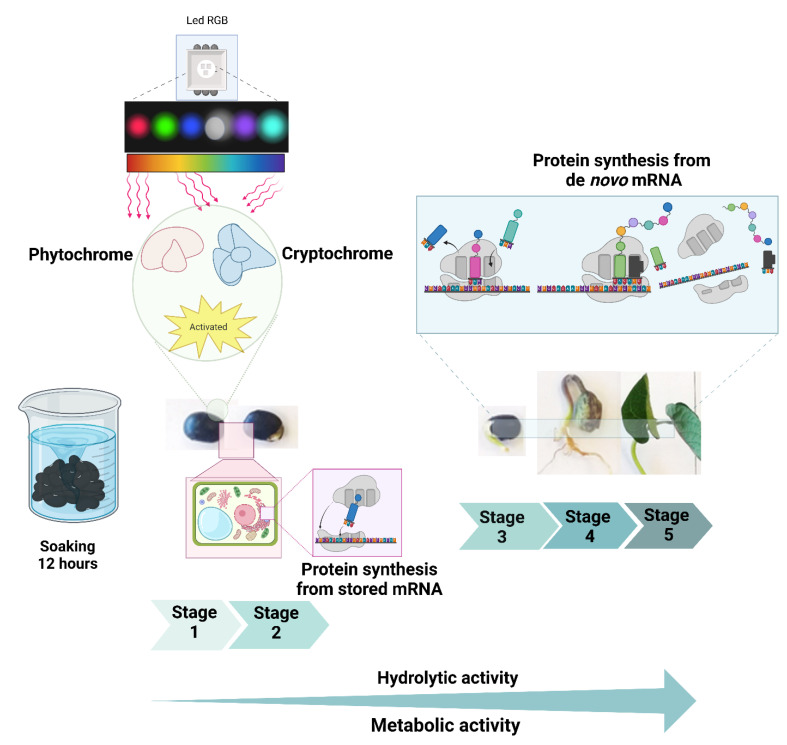
Protein activity of bean seed, as a result of seed soaking with water, as well as activation of cryptochromes and phytochromes, modulated by the color of emitted light (LED RGB) protein synthesis from stored messenger ribonucleic acid (mRNA) and from de novo mRNA.

Protein biosynthesis begins in the rough endoplasmic reticulum, continues in the smooth endoplasmic reticulum, and culminates in deposition within specialized organelles known as protein bodies, which are distributed throughout the seed, particularly in the embryo and cotyledons [34,36,37,38]. Once germination begins, stored proteins are hydrolyzed to release amino acids, which contribute to the synthesizing new proteins (e.g., de novo proteins) with diverse functions. These proteins are eventually degraded again to regenerate amino acids. This protein–amino acid cycle is essential for seed survival and subsequent plant development (Figure 3) [33,34,39].

Additionally, essential nutrients such as carbohydrates accumulate to serve as the primary energy source during germination [22,40]. This process also enhances nutrient profiles and bioavailability, improving protein digestibility, increasing antioxidant concentrations, and generating bioactive molecules such as peptides [6,41,42].

Drying of the sprouts is carried out to facilitate milling and prolong the shelf life of the final product (bean sprout flour). The drying temperature is important because it has been shown that the drying process at temperatures higher than 90 °C causes a decrease in protein solubility and in its techno-functional and sensory properties. At the same time, it has been reported that non-enzymatic browning (Maillard reaction) is subject to increased drying temperature. Based on the above, several authors have indicated that an optimum drying temperature for beans is 40 °C since, at this value, the nutritional quality is maintained and its preservation is guaranteed [43,44].

### 3.3. Protein Quantification During Germination

Protein concentration was determined by physiological changes occurring during the germination of black beans. The protein content of this seed ranges between 15 and 25% of its total composition. Proteins are classified into simple, conjugated, and derivative types [10,14]. The first group primarily consists of albumins, globulins, glutelins, and prolamins, with albumins and globulins being the most abundant in black beans [10,14,27,45]. These storage proteins play a key role in providing essential elements such as nitrogen, sulfur, and carbon, which are required for germination and subsequent plant development [27].

**Figure 4:**
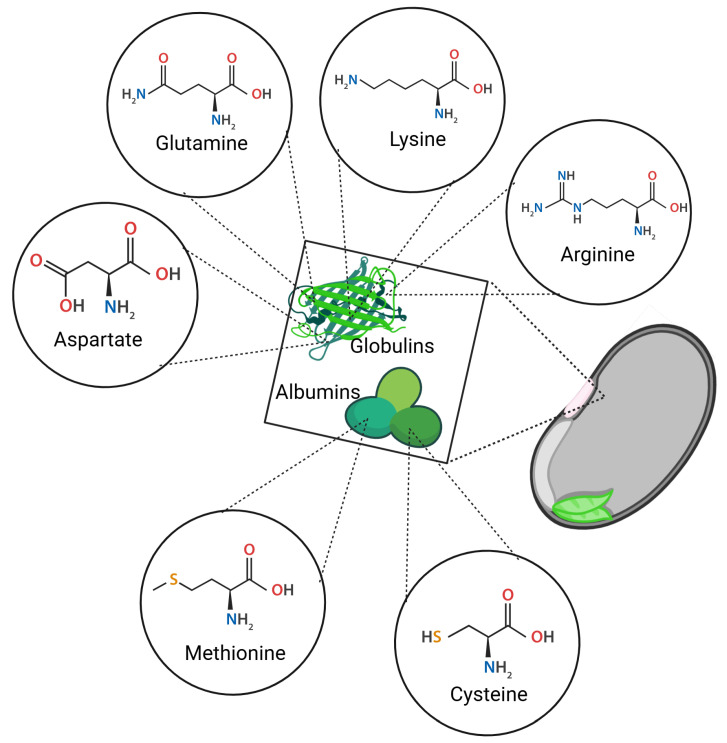
Main amino acids present in albumins and globulins of black beans.

From a nutritional perspective, albumins have higher sulfur-containing amino acids such as cysteine and methionine, whereas globulins exhibit higher concentrations of glutamine, aspartic acid, arginine, and lysine (Figure 4). Globulins, primarily storage proteins, constitute 35–72% of the total seed protein content [27,46]. As a reference, the initial concentrations of albumins and globulins in non-germinated seeds were quantified as 65.88 ± 6.89 mg/g and 74.23 ± 4.68 mg/g, respectively.

#### 3.3.1. Quantification of Albumins

**Figure 5:**
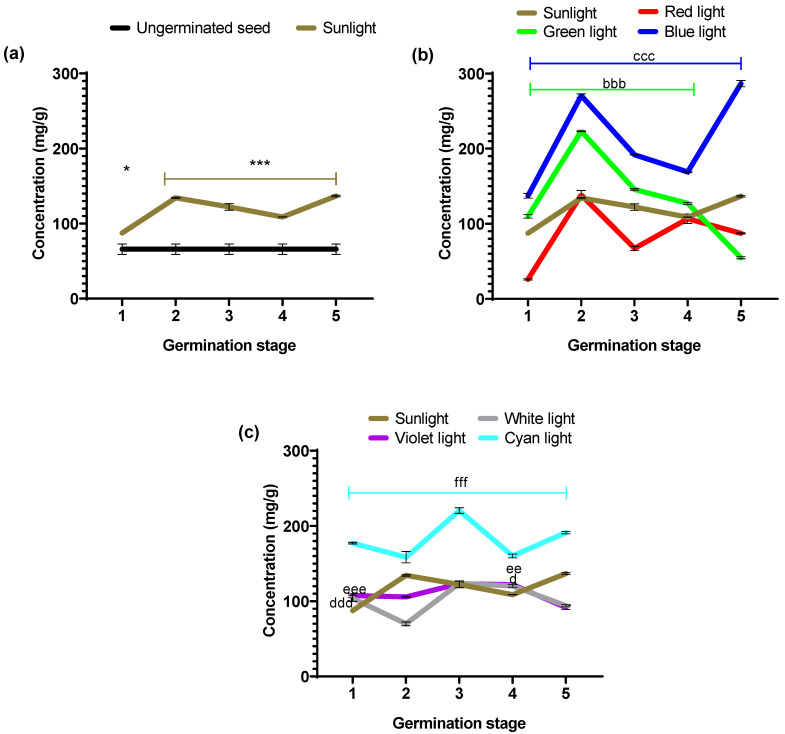
Albumin concentration obtained during the germination process performed with different light colors for the following: (**a**) ungerminated seed vs. germination with sunlight (daylight); (**b**) germination performed with the primary colors of the RGB LED system (red, green, and blue); and (**c**) germination performed with the colors resulting from the combination of RGB (white, cyan, and violet). Values are represented as mean ± SEM. n = 3, two-way ANOVA and post hoc Dunnett calculation done * *p* = 0.05 and *** *p* ˂ 0.0001 vs. ungerminated and bbb *p* ˂ 0.0001, ccc *p* ˂ 0.0001, d *p* = 0.05, ddd *p* ˂ 0.0007, ee *p* = 0.001, eee *p* ˂ 0.0001, fff *p* ˂ 0.0001 vs. sunlight.

Figure 5 shows the albumin concentrations obtained with each stage’s evaluated light colors. Table 1 shows the percentage values of increases and decreases calculated according to the control groups (ungerminated seed vs. sunlight and experimental groups vs. sunlight).

Significant variations in albumin concentration were observed in seeds exposed to different light colors during germination (Figure 5). The effect of each light color on the protein fraction content during black bean germination is unique and is associated with the seed’s dynamic metabolism and the influence of irradiated light color [26]. To determine the effect of the germination process on the bean seed, the first experimental condition evaluated was with sunlight, taking as a reference the seed without germination (negative control).

Figure 5a shows the concentrations of the albumin fraction of the ungerminated seed and those obtained under sunlight. The latter exhibited significantly higher concentrations than the ungerminated seed at all five stages (* *p* = 0.005 and *** *p* < 0.0001). In that sense, the albumin concentration increases in all five stages analyzed under sunlight compared to non-germinated seeds. Particularly, stages 2 and 5 of the sunlight group showed the highest percentage increases (Table 1).

Figure 5b shows albumin concentration in seeds germinated under the primary light colors of the RGB system (red, green, and blue) and sunlight as a positive control. The albumin content for the seeds growing under monochromatic light illumination showed different behavior compared to the growing under sunlight. However, the RGB illumination showed increases and decreases in albumin concentration between the different stages; red light did not improve the albumin content.

It is worth mentioning that green and blue light produces albumin concentrations higher than sunlight between stages 1 to 4. The blue light produced an increase in all five stages, reaching as high as 286.58 ± 4.28 mg/g in stage 5.

Red light showed a statistically significant decrease (*p* < 0.001) in albumin concentrations in stages 1, 3, and 5. In contrast, stages 2 and 4 showed no significant difference compared to the concentration obtained with sunlight (*p* = 0.5671 and *p* = 0. 9130, respectively). Green light showed statistically significantly higher concentration values (bbb *p* < 0.0001) in stages 1 to 4; only in stage 5 was the concentration lower (*p* < 0.0001) than that obtained under sunlight conditions. On the other hand, blue light exhibited statistically significant higher concentration values (ccc *p* < 0.0001) during the five stages of the germination process, in contrast to the values for germination under sunlight conditions.

In Figure 5c, we plotted the albumin concentration in seeds germinated under white, violet, and cyan LED illumination, taking as a positive control sunlight germination. Illumination by white and violet light produces a concentration of albumins that is less or equivalent to that obtained with sunlight. For this fraction (albumins), these light sources do not have a benefit for germination other than that obtained by the classical germination process under sunlight. However, the cyan light source increased albumin content, similar to all germination stages. Thus, this is a more stable germination effect over time.

In the statistical point of view, these colors showed significant values above those obtained under sunlight at stages 1 (ddd *p* < 0.0007 and eee *p* < 0.0001) and 4 (d *p* < 0.05 and ee *p* < 0.001), respectively. In contrast, cyan light exhibited statistically significant higher values (fff *p* < 0.0001) during the five stages of the germination process with respect to sunlight.

On average, for the five stages, the cyan illumination produced an increment of albumin content of 181.47 ± 6.30 (mg/g), which is similar to the average produced by the blue light of 211.04 ± 15.54 (mg/g), meaning that it is possible to propose different strategies to produce functional foods of high albumin contents in certain germination stages like that obtained with the blue illumination for the early stage of non-visible radicle and the late seedling stage or a high–moderate albumin content in all germination stages with cyan light.

In Table 1, the experimental groups with statistically significant percentage increases during the different germination stages with respect to sunlight are described as follows: green light (1–4), blue light (1–5), white and violet light (1 and 4), and finally, cyan light (1–5).

#### 3.3.2. Quantification of Globulins

**Figure 6:**
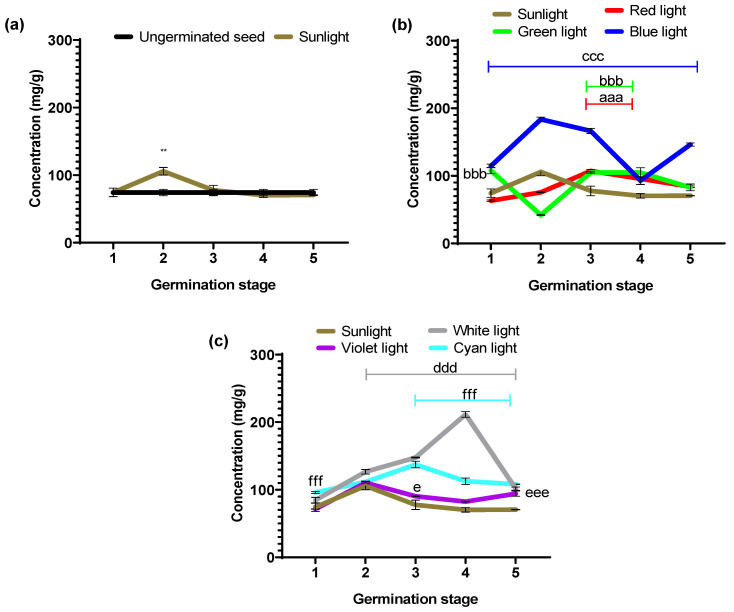
Globulin concentration obtained during the germination process performed with different light colors for the following: (**a**) ungerminated seed vs. germination with sunlight (daylight); (**b**) germination performed with the optical primary colors of the RGB LED system (red, green, and blue); and (**c**) germination performed with the colors resulting from the combination of RGB (white, cyan, and violet). Values are represented as mean ± SEM, n = 3. Two-way ANOVA and post hoc Dunnett. ** *p* = 0.001 vs. ungerminated. aaa *p* ˂ 0.0001, and bbb *p* ˂ 0.0001, ccc *p* ˂ 0.0001, ddd *p* ˂ 0.0007, e *p* = 0.001, eee *p* ˂ 0.0001, fff *p* ˂ 0.0001 vs. sunlight.

Figure 6 shows the globulin concentrations quantified during germination with different light colors. Table 2 shows the percentage values of increases and decreases calculated according to the control groups (ungerminated seed vs. sunlight and experimental groups vs. sunlight).

Figure 6a shows globulin concentrations of ungerminated and germinated seeds under sunlight. Germination under sunlight showed only in stage 2, a statistically significant (** *p* = 0.001) higher value in the concentration of the globulin fraction; for the remaining stages, the concentrations did not differ significantly from the non-germinated seeds.

Figure 6b shows the concentration values of the globulin fraction in seeds germinated under the primary light colors of the RGB system (red, green, and blue) and sunlight as a positive control. The globulin content of seeds germinated under monochromatic light showed different behaviors for each of the colors, resulting in increases and decreases in concentration during the five germination stages. It is worth mentioning that blue light produces a higher globulin concentration than sunlight, reaching a maximum value of 183.60 ± 3.39 mg/g in stage 2.

Red light showed no significant difference in globulin concentration compared to sunlight at stages 1 and 5; on the other hand, stage 2 showed a statistically significant lower concentration (*p* < 0.0001), while stages 3 and 4 showed statistically significant higher values (*p* < 0.0001). Green light exhibited significantly higher concentration values than those obtained under sunlight at stages 1, 3, and 4 (*p* < 0.0001), while stage 2 showed a significantly lower value (*p* < 0.0001), and stage 5 did not differ significantly from sunlight.

Exposure to blue light resulted in significantly higher concentrations (*p* < 0.0001) at all germination stages compared to sunlight values.

In Figure 6c, we plot the globulin concentration in seeds germinated under white, violet, and cyan LED illumination, taking sunlight germination as a positive control. White light showed no significant difference at stage 1; however, stages 2 to 5 showed significantly higher values (ddd *p* < 0.0001) relative to sunlight (Figure 6c). Violet light showed no significant difference in concentration at stages 1, 2, and 4, while stages 3 and 5 showed statistically higher values (e *p* = 0.0424 and eee *p* < 0.0001), respectively, compared to sunlight values. Exposure to cyan light resulted in statistically significant higher concentration values in stages 1, 3, 4, and 5 (*p* < 0.0001), while stage 2 did not differ significantly from the values obtained under sunlight.

On average, the highest globulin concentrations (mg/g) were observed with blue light 140.77 ± 8. 96, white light 134.39 ± 11.84, and cyan 113.21 ± 3.79. These can contribute to developing products with high globulin content and techno-functional applications obtained from a vegetable source.

In Table 2, the experimental groups with statistically significant percentage increases during the different germination stages compared to sunlight are described as follows: red light stage (3–5), green light (1, 3–5), blue light (1–5), white light (2–5), violet light (4 and 5), and finally, cyan light (1, 3–5).

Light is fundamental in seed growth and development regulation, promoting physiological and metabolic modifications, and modulating gene expression [47]. Spectral variability in intensity, duration, and wavelength combination provides optimal conditions for enhancing germination and seed properties, including yield, morphology, nutritional and chemical composition [48].

Seeds possess photoreceptors capable of capturing a broad spectrum of wavelengths, triggering adaptive responses through the activation of signal transduction pathways [35]. The influence of light on black bean seeds is associated with the excitation of cryptochromes, phytochromes, phototropins, and chlorophyll a and b [48], triggering internal signals that regulate germination.

RGB LED light sources offer several advantages over other lighting systems, including low cost, compact design, long lifespan, minimal heat generation, and the ability to generate various wavelengths (400–700 nm) within the electromagnetic spectrum, a range known to encompass photosynthetically active radiation [48,49].

The impact of light on germinating cells promotes increased cell multiplication rather than elongation. Additionally, light stimulates the production of growth-promoting substances such as GA [50]. The observed fluctuations in albumins and globulin concentration under different light colors can be explained by the seed’s physiological demands.

The increase in albumins and globulins at stage 2 may be attributed to synthesizing hydrolytic enzymes responsible for biopolymer degradation, facilitating tissue softening and radicle emergence at stage 3 [22,33,51].

An increase in albumin concentration is linked to the protective needs of seeds and seedlings against pathogens such as viruses, bacteria, and fungi. Research has shown that certain peptides within this fraction, particularly 2S albumins, exhibit bactericidal and fungicidal properties. They achieve this by destabilizing the cell membranes of these microorganisms through charge differences [52,53,54].

During germination, several enzymes dispersed throughout the cell, such as carboxypeptidase and proteinase, are involved [38]. The latter exhibits optimal functioning when the mobilization of proteins and amino acids from the cotyledon to the peripheral tissues occurs more rapidly to produce more enzymes and structural proteins [55,56,57].

The degradation of seed reserves occurs in response to embryonic axis signaling to the cotyledons [58]. Storage proteins within cotyledons are hydrolyzed during germination, leading to radicle and root emergence [55].

At the same time, it has been reported that exposure to blue light is capable of suppressing growth in stems and leaves but increasing the concentration of molecules with bioactive potential; on the other hand, the combination of different wavelengths can exert a synergistic or antagonistic effect on germination [59].

### 3.4. Correlation Analysis Between Albumins and Globulin Concentration

**Figure 7:**
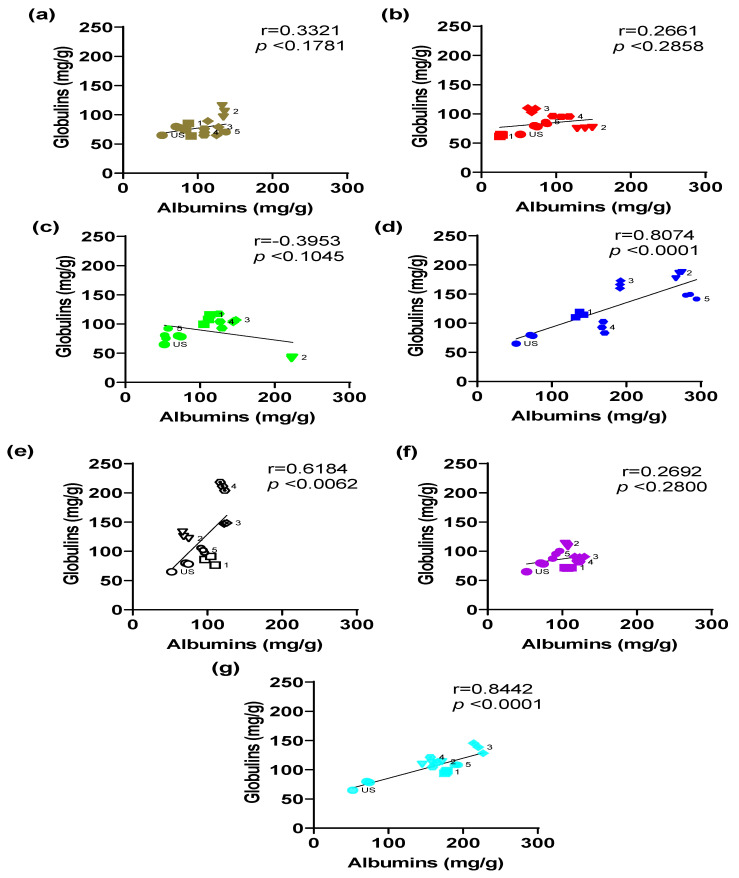
Correlations between albumins and globulin concentrations obtained during black bean germination under different light colors: (**a**) sunlight (daylight), (**b**) red light, (**c**) green light, (**d**) blue light, (**e**) white light, (**f**) violet light, and (**g**) cyan light. Values represented as mean ± SEM. Correlations were performed with Pearson’s r. US: ungerminated seed. 1–5 germination stages.

To relate the concentrations of both protein fractions, a correlation analysis was performed for each light color evaluated during the germination process. The correlation analysis between albumins and globulin concentrations in black bean sprouts exposed to different light colors and evaluated at different developmental stages revealed considerable variability depending on the wavelengths used (Figure 7). These results reflect the complex interaction between light color and the metabolic regulation proteins during germination.

Under sunlight conditions, the correlation was positive, without statistical significance (r = 0.3321, *p* = 0.1781); however, both fractions showed their highest concentration values at stage 2 (Figure 7a). Light, specifically sunlight, is an important factor in the metabolic regulation of proteins during germination. It is composed of different wavelengths, and it depends on geographical location, time of year, and climate, which can lead to variable physiological responses [60].

Red-light treatment showed a positive correlation without statistical significance (r = 0.2661, *p* = 0.2858), with its highest concentration value at stage 3 (Figure 7b). This indicates that this wavelength has no relationship between the fractions, which agrees with research that associates red light mainly with photomorphogenic processes [61,62].

In contrast, green light showed a negative correlation (r = −0.3953, *p* = 0.1045), although not statistically significant, with stage 1 and 4 increments (Figure 7c). However, this negative trend is consistent with studies suggesting that green light can generate variable responses by exerting photoreceptor-independent and photoreceptor-dependent effects; at the same time, it has been reported to possess inhibitory behavior against responses elucidated for blue light, e.g., stomatal opening and consecutive signaling cascades [63,64]. It is thus attributed to an atypical regulation during the process of photomorphogenesis [65].

Violet light showed a nonsignificant positive correlation (r = 0.2692, *p* = 0.2800), with increases in both proteins in stage 2 (Figure 7f). Although violet light has been less studied compared to the other colors, the results obtained in the concentrations and correlation analysis suggest that this color does not exert a beneficial effect on germination compared to germination performed with sunlight or white light, as described by some authors [66].

Finally, the most relevant and significant correlations were observed in sprouts exposed to blue light (r = 0.8074, *p* < 0.0001), white light (r = 0.6184, *p* = 0.0062), and cyan light (r = 0.8442, *p* < 0.0001) (Figure 7d, Figure 7e and Figure 7g, respectively). The highest increases in both proteins occurred in stages 2, 4, and 3, respectively.

The behavior exhibited in the correlation analysis for each color is influenced by the behavior of each protein fraction during the five stages of the germination process. The positive and significant correlations suggest that the short wavelengths (blue and cyan) and the full spectrum (white light) can present a joint regulation that improves the protein content in the sprouts.

These findings highlight that light color is a key factor in the dynamics between albumins and globulins during germination, with blue, cyan, and white lights being the most effective in promoting a positive and significant correlation between these protein fractions. This reinforces the importance of light as a metabolic modulator, consistent with previous literature on photobiology and light quality in agricultural systems [67].

This study demonstrates that exposure to different colors of LED light during black bean germination has a relationship with the protein metabolism of this seed and under certain lighting colors, the protein content can be increased compared to germination under solar conditions. This finding is relevant from both physiological and nutritional points of view since albumins and globulins represent the main protein fractions in legumes and play a key role in the nutritional and functional quality of germinated beans [68].

The results suggest that blue light activates photoreceptors such as cryptochromes and phototropins, which may directly influence protein synthesis and degradation during black bean germination, which is consistent with other published work [62,69,70,71].

These results open new perspectives to optimize the production of sprouts of high nutritional value by specific control of light color, which could contribute to the design of functional foods enriched with high-quality proteins, essential in plant-based diets, and strategies to combat malnutrition [72].

The consumption of black bean sprouts obtained under blue, cyan and white light sources could offer a more bioavailable source of essential proteins, in addition to bioactive compounds associated with sprouting, such as peptides with antihypertensive, antioxidant and anti-inflammatory properties [73].

This approach, which integrates applied photobiology with functional nutrition, represents an innovative strategy to improve the protein quality of sprouts, contributing to the development of more sustainable functional foods adapted to the nutritional needs of vulnerable populations [74].

## 4. Conclusions

The germination process under environmental conditions influences the amount of protein by increasing or maintaining albumins and globulin concentrations, respectively, in contrast to ungerminated or dormant seeds. By performing the process under different colored lighting conditions sources (RGB LED system), each color exhibits unique behavior during the whole germination development of the bean seed.

The germination process is a multifactorial phenomenon in which the seed’s viability, adaptive capacity to changes in the environment, water absorption (soaking and imbibition), and activation and interaction with light result in a dynamic metabolism. Changes in albumin and globulin concentrations suggest an adaptive response to environmental factors such as light. This indicates the importance of this factor in regulating protein expression during germination.

Among the colors evaluated, blue and cyan light colors promote the albumin content, and blue, white, and cyan for globulins stand out, as they showed the highest percentages of increase in at least four stages. For both fractions, the blue light color exhibited the best results.

The increase in the concentration of the fractions studied in this work highlights a fact about the protein of this legume, and that is that germination itself is a cost-effective process capable of improving the amount of these macromolecules; performing this with specific light colors such as blue, white, and cyan, improves, even more, the amounts of these macromolecules.

In other words, although black beans are considered a functional food because of their multiple nutritional benefits (carbohydrates, dietary fiber, secondary metabolites such as phenolic compounds and protein), germination under specific light colors can increase this functionality.

On the other hand, the germination process is a viable alternative that increases protein content, meeting the growing demand for animal protein substitution in scenarios where access to meat is limited or not desired.

The application of bean sprouts as flour can be related to the development of new and improved foods with a high protein content, for example, in the production of pasta and bakery products or nixtamalization. In addition, protein isolates and concentrates can be obtained, useful in the production of food supplements.

Future research will study the effect of intrinsic light variables such as intensity, power, and photoperiods with different colors on seed germination. At the same time, we intend to evaluate germination from a rheology point of view, specifically, how mechanical properties such as viscosity, elasticity, and tissue permeability evolve during water absorption and embryo growth. By integrating machine learning with rheological data (e.g., stress–strain curves, hydration kinetics), we aim to model the dynamic interaction between embryo expansion and seed coat strength. Then, predictive algorithms will correlate these mechanical changes with germination success, enabling specific strategies to improve crop performance.

## Figures and Tables

**Table 1:** Effect of light color illumination on albumins expressed as percentage increase and decrease during black bean germination.

	Negative Control	Positive Control		Experimental Groups					
	A			B					
Germination Stage	US	Sunlight		Red Light	Green Light	Blue Light	White Light	Violet Light	Cyan Light
1	0	32.73 ± 3.22 *	0	−70.25 ± 1.00 ^a^	25.63 ± 2.71 ^ab^	56.90 ± 3.51 ^abc^	18.70 ± 4.62 ^abd^	22.99 ± 2.64 ^abd^	102.57 ± 1.12 ^acdef^
2	0	103.78 ± 1.37 *	0	3.17 ± 4.33	66.21 ± 0.39 ^ab^	101.58 ± 1.80 ^abc^	−47.95 ± 1.87 ^abcd^	−21.24 ± 0.76 ^abcde^	18.02 ± 5.62 ^abcdef^
3	0	85.48 ± 6.64 *	0	−44.97 ± 2.33 ^a^	19.23 ± 0.95 ^ab^	56.95 ± 0.24 ^abc^	1.20 ± 0.95 ^bcd^	0.82 ± 3.03 ^bcd^	80.45 ± 3.00 ^abcdef^
4	0	65.05 ± 0.21 *	0	−1.90 ± 5.79	16.90 ± 1.09 ^ab^	55.44 ± 0.92 ^abc^	10.52 ± 1.59 ^abd^	12.23 ± 1.02 ^abd^	47.25 ± 2.33 ^abcef^
5	0	107.47 ± 1.62 *	0	−36.19 ± 0.48 ^a^	−59.94 ± 0.99 ^ab^	109.67 ± 3.06 ^abc^	−31.30 ± 1.03 ^acd^	−32.98 ± 1.91 ^acd^	39.86 ± 1.13 ^abcdef^

Values calculated according to Equation (1), represented as mean ± SEM, n = 3. Two-way ANOVA and Post hoc Tukey. A: Comparison between US group and sunlight conditions; B: comparisons between experimental groups and sunlight conditions. * *p* < 0.05 vs. US. ^a^ *p* < 0.05 vs. sunlight. ^b^ *p* < 0.05 vs. red light. ^c^ *p* < 0.05 vs. green light. ^d^ *p* < 0.05 vs. blue light. ^e^ *p* < 0.05 vs. white light. ^f^ *p* < 0.05 vs. violet light. US: ungerminated seed.

**Table 2:** Effect of light color illumination on globulins expressed as percentage increase and decrease during black bean germination.

	Negative Control	Positive Control		Experimental Groups					
	A			B					
Germination Stage	US	Sunlight		Red Light	Green Light	Blue Light	White Light	Violet Light	Cyan Light
1	0	0.19 ± 8.39	0	−14.92 ± 1.31	44.85 ± 6.33 ^ab^	54.15 ± 3.62 ^ab^	13.62 ± 5.86 ^bcd^	−3.77 ± 0.33 ^cde^	29.19 ± 1.81 ^abdf^
2	0	42.42 ± 7.60 *	0	−28.53 ± 0.49 ^a^	−60.20 ± 0.83^ab^	73.66 ± 3.21 ^abc^	19.82 ± 3.14 ^abcd^	4.38 ± 1.52 ^bcd^	5.58 ± 1.19 ^bcd^
3	0	4.70 ± 9.45	0	37.86 ± 2.92 ^a^	35.15 ± 1.39 ^a^	114.11 ± 4.77 ^abc^	90.02 ± 0.63 ^abcd^	16.28 ± 0.40 ^bcde^	76.79 ± 6.39 ^abcdf^
4	0	−5.32 ± 4.50	0	36.05 ± 0.50 ^a^	49.26 ± 10.16 ^a^	32.24 ± 7.99 ^ac^	200.70 ± 5.88 ^abcd^	17.34 ± 0.98 ^abce^	60.34 ± 6.91 ^abdef^
5	0	−4.88 ± 0.45	0	18.93 ± 1.61 ^a^	17.39 ± 7.01 ^a^	107.15 ± 3.42 ^abc^	44.18 ± 3.43 ^abcd^	33.54 ± 5.39 ^ad^	53.38 ± 0.35 ^abcdf^

Values calculated according to Equation (1), represented as mean ± SEM, n = 3. Two-way ANOVA and Post hoc Tukey. A: Comparison between US group and sunlight conditions; B: comparisons between experimental groups and sunlight conditions. * *p* < 0.05 vs. US. ^a^ *p* < 0.05 vs. sunlight. ^b^ *p* < 0.05 vs. red light. ^c^ *p* < 0.05 vs. green light. ^d^ *p* < 0.05 vs. blue light. ^e^ *p* < 0.05 vs. white light. ^f^ *p* < 0.05 vs. violet light. US: ungerminated seed.

## Data Availability

The original contributions presented in this study are included in the article. Further inquiries can be directed to the corresponding author.
